# Comprehensive assessments of germline deletion structural variants reveal the association between prognostic MUC4 and CEP72 deletions and immune response gene expression in colorectal cancer patients

**DOI:** 10.1186/s40246-020-00302-3

**Published:** 2021-01-11

**Authors:** Peng-Chan Lin, Hui-O Chen, Chih-Jung Lee, Yu-Min Yeh, Meng-Ru Shen, Jung-Hsien Chiang

**Affiliations:** 1 Department of Computer Science and Information Engineering, College of Electrical Engineering and Computer Science, National Cheng Kung University, Tainan, Taiwan; 2 Institute of Medical Informatics, National Cheng Kung University, Tainan, Taiwan; 3 Department of Oncology, National Cheng Kung University Hospital, College of Medicine, National Cheng Kung University, Tainan, Taiwan; 4 Department of Internal Medicine, National Cheng Kung University Hospital, College of Medicine, National Cheng Kung University, Tainan, Taiwan; 5 Graduate Institute of Clinical Medicine, College of Medicine, National Cheng Kung University, Tainan, Taiwan; 6 Department of Obstetrics and Gynecology, National Cheng Kung University Hospital, College of Medicine, National Cheng Kung University, Tainan, Taiwan; 7 Department of Pharmacology, National Cheng Kung University Hospital, College of Medicine, National Cheng Kung University, Tainan, Taiwan

**Keywords:** Whole-genome sequencing, Cancer risk, Deletion structural variants, *MUC4*, *CEP72*

## Abstract

**Background:**

Functional disruptions by large germline genomic structural variants in susceptible genes are known risks for cancer. We used deletion structural variants (DSVs) generated from germline whole-genome sequencing (WGS) and DSV immune-related association tumor microenvironment (TME) to predict cancer risk and prognosis.

**Methods:**

We investigated the contribution of germline DSVs to cancer susceptibility and prognosis by silicon and causal inference models. DSVs in germline WGS data were generated from the blood samples of 192 cancer and 499 non-cancer subjects. Clinical information, including family cancer history (FCH), was obtained from the National Cheng Kung University Hospital and Taiwan Biobank. Ninety-nine colorectal cancer (CRC) patients had immune response gene expression data. We used joint calling tools and an attention-weighted model to build the cancer risk predictive model and identify DSVs in familial cancer. The survival support vector machine (survival-SVM) was used to select prognostic DSVs.

**Results:**

We identified 671 DSVs that could predict cancer risk. The area under the curve (AUC) of the receiver operating characteristic curve (ROC) of the attention-weighted model was 0.71. The 3 most frequent DSV genes observed in cancer patients were identified as *ADCY9*, *AURKAPS1*, and *RAB3GAP2* (*p* < 0.05). The DSVs in *SGSM2* and *LHFPL3* were relevant to colorectal cancer. We found a higher incidence of FCH in cancer patients than in non-cancer subjects (*p* < 0.05). *SMYD3* and *NKD2DSV* genes were associated with cancer patients with FCH (*p* < 0.05). We identified 65 immune-associated DSV markers for assessing cancer prognosis (*p* < 0.05). The functional protein of *MUC4* DSV gene interacted with *MAGE1* expression, according to the STRING database. The causal inference model showed that deleting the *CEP72* DSV gene affect the recurrence-free survival (RFS) of *IFIT1* expression.

**Conclusions:**

We established an explainable attention-weighted model for cancer risk prediction and used the survival-SVM for prognostic stratification by using germline DSVs and immune gene expression datasets. Comprehensive assessments of germline DSVs can predict the cancer risk and clinical outcome of colon cancer patients.

**Supplementary Information:**

The online version contains supplementary material available at 10.1186/s40246-020-00302-3.

## Introduction

Large-scale germline structural variants, especially deletion structural variants (DSVs), can affect gene expression with a partial or complete loss of gene function and increased cancer risk in patients [1, 2]. Several studies have reported the germline pathogenic DSVs through whole-genome sequencing (WGS) [3, 4]. For example, patients with germline *RAD51C* exon 5 deletion or *ATM* exon 9 deletion were confirmed as having hereditary cancer syndrome [5]. Instead of multiplex ligation-dependent probe amplification (MLPA) and next-generation sequencing (NGS) panels, WGS with multiple cancer-associated DSVs has become more widely used for cancer risk assessment. However, the role of germline DSVs and DSV immune-related association tumor microenvironment (TME) in cancer risk and prognosis had not been sufficiently understood.

To investigate the contribution of germline DSVs to cancer susceptibility and prognosis, we used silicon and causal inference models. Prediction models are important when classifying individuals for predicting the risk and survival stratification to minimize the impact of cancer and optimize treatment [6]. The application of machine learning techniques, such as deep learning (DL) and inherited risk genomic variation analysis, is rapidly developing [7, 8]. As DL has improved the ability to predict inherited cancer genomic susceptibility, we focused on DL as an attention-weighted model with multilayer perceptrons (MLPs) [9], which can reveal the importance of each DSV for predicting cancer risk. Additionally, we used the survival support vector machine (survival-SVM) for selecting the features of prognostic DSVs.

Here we describe the prediction model of germline DSVs in cancer patients with and without family cancer history (FCH). We used a machine learning model for survival stratification to assess the prognosis and demonstrated the biological relevance of germline DSVs and TME-related immune gene expression.

## Results

### Germline DSV detection from whole-genome sequencing

**Fig. 1:**
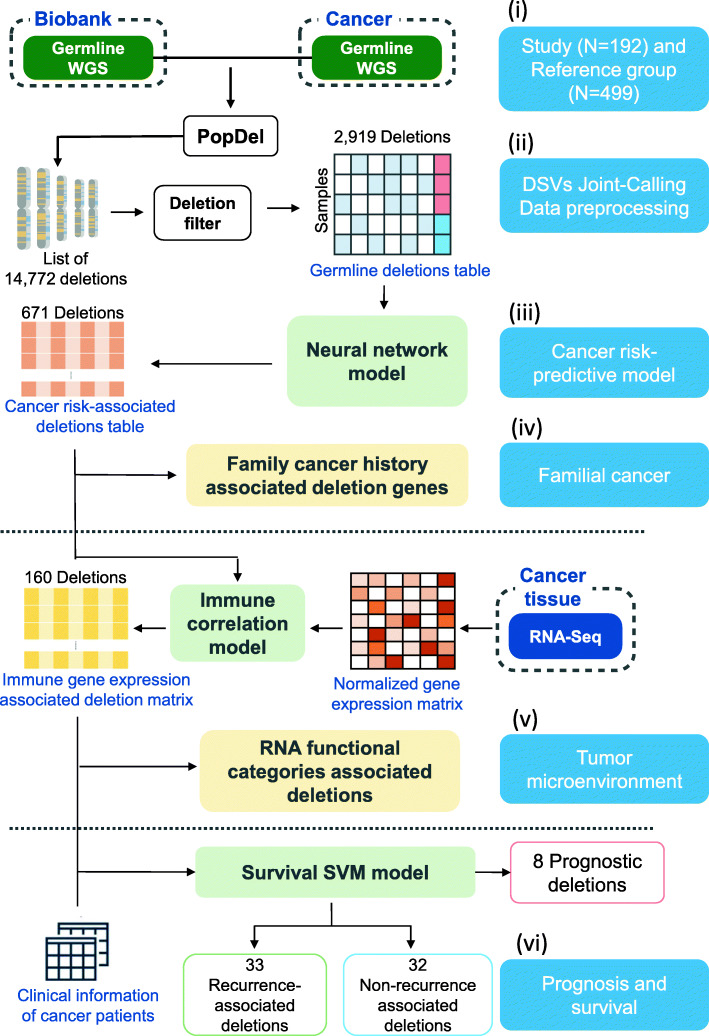
Study design and workflow. Study design and overall workflow of WGS analysis of germline DSVs and immune gene expression for cancer risk prediction and survival stratification. In total, 192 cancer patients **(i)**—comprised of 120 with colorectal cancer, 29 with endometrial cancer, 35 with ovarian cancer, and eight with breast cancer—were enrolled in the study group, and 499 non-cancer subjects (i) were included in the reference group. Genomic data, including WGS, gene expression, clinical outcome, and FCH, were collected. First, we used the PopDel method (ii) to detect DSVs and perform data preprocessing (ii) from the WGS analysis of all subjects. The cancer risk predictive model (iii) was built with an attention-weighted model. We also studied DSVs in familial cancer (iv). Second, we examined the relationship between DSVs and the tumor microenvironment (v). Immune gene expression data were normalized. We constructed an immune gene expression-associated DSV correlation matrix with the point-biserial correlation. Third, a machine learning method with a survival support vector machine (survival-SVM) and Kaplan–Meier survival analysis was applied to examine prognosis and survival (vi)

We applied feature extraction and selection methods to analyze genomics data for the detection of cancer-associated, immune-associated, and prognosis-associated DSVs. (Fig. 1).We utilized the PopDel [10] tool to detect germline DSVs. The WGS data of cancer patients and non-cancer subjects were input simultaneously for joint calling. A total of 14,772 autosomal DSVs with sizes ranging from 500 to 10,000 base pairs were called simultaneously across all samples. We focused our analysis on DSVs occurring in at least 1% of the samples of both cancer and non-cancer populations at minor allele frequency (MAF) above 5% [11]. A total of 2919 DSVs that passed the filtering criteria were further used to build a classification model.

### Predicting cancer risk with whole-genome DSVs and MLP

**Fig. 2:**
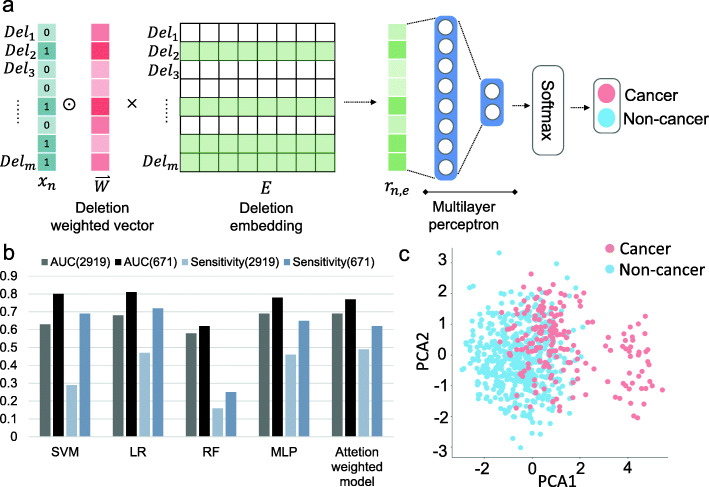
Feature selection of DSVs to distinguish cancer and non-cancer subjects. **a** The architecture of the attention-weighted model for selecting the cancer risk DSV features. The primary purpose was to classify cancer or non-cancer subjects by the neural network. This was a MLP model based on the attention mechanism. We used *n* samples (*x*_*n*_) as input in the attention-weighted model: every sample had *m* (*Del*_*m*_) filtered deletions. A value of 1 in the deletion vector indicates that the sample has the specific deletion, while 0 implies no deletion. A weighted vector ($\underset{W}{\to }$) is associated to the input layer to identify the importance of each deletion (red color gamut). Additionally, an embedding layer (*E* represents the embedding table, *e* denotes the embedding size) is applied to reduce the feature size and each deletion. We took the sum of each column and obtained a vector that can represent the information of the input deletion features (*r*_*n*, *e*_); this is the input of multilayer perceptron. The output of MLP utilizes the SoftMax layer. The output labels are cancer or non-cancer subjects. **b** Performance of five machine learning strategies (attention-weighted model, MLP SVM, RF, and LR) for cancer risk prediction with different number of features (2919 and 671 cancer-associated DSVs). The attention-weighted model was more sensitive (AUC = 0.71, sensitivity = 0.57) than the other methods. All of the models performance are improved with 671 cancer-associated DSVs. **c** PCA plot by cancer-associated DSVs. Red dots represent cancer subjects, and blue dots represent non-cancer subjects. A total of 671 cancer-associated DSVs with positive weights were used for PCA. DSVs can distinguish cancer and non-cancer subjects

Germline genomic DSVs are known to be associated with increased risk for cancer [12], and several studies have reportedly applied machine learning tools for developing prediction models [13]. To learn the importance of each DSV for classifying cancer or non-cancer samples, we consider the attention-weighted model to be the final approach. Furthermore, the attention-weighted model had the best performance to predict cancer risk. Herein, several machine learning strategies for classification were applied and evaluated. We used an SVM with linear kernel and logistic regression (LR), both of which were well-known linear models. We also used random forests (RF) to test nonlinear results. Moreover, neural network strategies, such as multilayer perceptron and attention-weighted models, were also adopted (Fig. 2a).

The area under the curve (AUC) from the receiver operating characteristic curve (ROC) and the performance of models (i.e., sensitivity) are crucial for clinical use. Among these methods, the attention-weighted model (AUC = 0.71, sensitivity = 0.58) performed the best with 2919 DSVs (Fig. 2b and Supplementary Fig. 1). All of the model’s performances were improved with 671 cancer-associated DSVs. In total, 671 of 2919 significant DSVs were selected for the prediction of cancer risk with positive weights from the attention-weighted model (Supplementary Table 1A). There were no demographic biases in the population data (Supplementary Fig. 2). The size and distribution of deletions on each chromosome were no different between cancer patients and non-cancer subjects (Supplementary Fig. 3). The cancer and non-cancer samples could be distinguished with 671 DSVs in principal components analysis (PCA). The 671 DSVs were divided into two clusters by using hierarchical clustering. We used REACTOME [14] to perform pathway enrichment of genes. There were 92 genes in the first cluster and 125 genes in the second cluster. In the first cluster, there were 11 genes (*MUC17*, *MUC19*, *MUC4*, *MUC6*, *GALNT9*, *B3GNTL1*, *KCNMB2*, *UNC13B*, *RIMS1*, *SMYD3*, *MYT1*) that enriched 19 significant pathways (*p* value < 0.05) which were relevant to O-linked glycosylation of mucins, Neurotransmitter Release Cycle, defective factor causing familial hyperphosphatemic tumoral calcinosis, etc. In the second cluster, there were 27 genes (*KCNJ6*, *ADCY9*, *GNG2*, *ADCY8*, *PFKFB3*, *ESR1*, *MUC12*, *MUC4*, *GALNT9*, *B3GNTL1*, *ATP13A4*, *ATP2B4*, *ATP11A*, *SBF2*, *ADAP1*, *F11*, *CACNG7*, *KCNIP1*, *CACNA1C*, *RPTOR*, *PSMA8*, *ACTN2*, *PPP6R2*, *CYP4F11*, *CYP4F12*, *SOD2*) that enriched 47 significant pathways (*p* value< 0.05) which involved defective factor causing hereditary angioedema, Ion transport by P-type ATPases, diseases of hemostasis, etc. (Supplementary Table 2). However, there were no differences between each type of cancer (Supplementary Fig. 4A). Further analyses were conducted to determine the genes with DSVs and relevant pathways related to different cancer types. The result indicated that the DSVs in *SNTG2*, *PCMT1*, *DACT2*, *CBX3*, *ATP11A*, and *SHC2* were associated with breast cancer. DSVs in *SGSM2* and *LHFPL3* were relevant to colorectal cancer, whereas *ADAP1*, *DLGAP2*, *ERC1*, and *PPP6R2* were related to gynecologic cancer (Supplementary Fig. 4B and C).

### The mutational landscape of DSVs and their significance in familial cancer patients

**Fig. 3:**
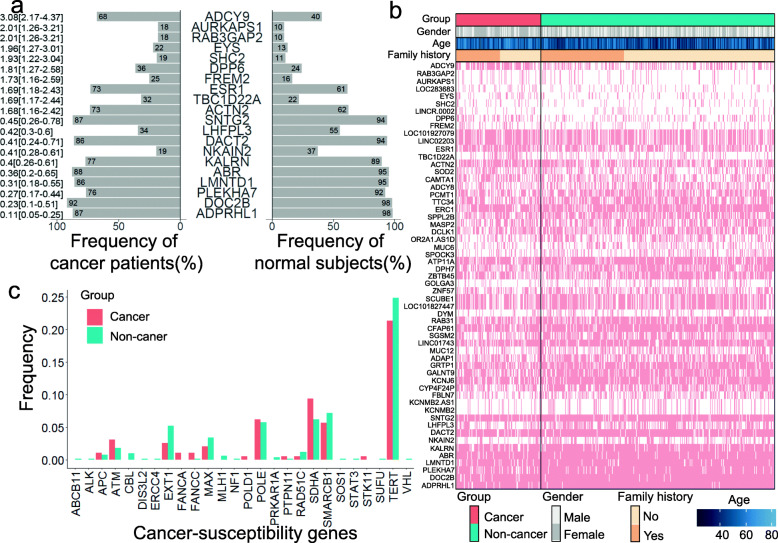
The frequency spectrum of DSVs in cancer and non-cancer subjects and germline cancer susceptibility gene analysis. **a** Bar plot of the top 10 DSV genes with significant odds ratios and *p*-adjusted values < 0.05 by the false discovery rate (FDR) in the cancer group and non-cancer group separately. The *x*-axis indicates the percentage of subjects who carry DSV genes, while the *y*-axis represents the DSV genes. **b** Heatmap of 57 DSV genes and clinical information. Genes with an odds ratio and *p*-adjusted value < 0.05 by the FDR were selected. The clinical information includes sex, age, and FCH. **c** Bar plot of 26 DSV genes intersected in 565 germline cancer susceptibility genes in cancer and non-cancer subjects. The *x*-axis indicates the well-known cancer susceptibility genes, and the *y*-axis indicates the frequency of the genes in cancer and non-cancer subjects. *FANCA*, *POLD1*, and *STK11* gene deletions occurred only in the cancer group

The germline mutational landscape of DSVs plays an important role in cancer patients with or without FCH. Patients who have one or more blood relatives within third-degree suffering from any types of cancer are considered having family cancer history. The odds ratios were estimated to identify which genes with DSVs were associated with FCH. We chose the top 10 DSV genes associated with an increased risk of cancer (odds ratios (OD) > 1) and the bottom 10 DSV genes associated with a decreased risk of cancer (OD< 1) from 671 DSVs associated with cancer risk (Fig. 3a). The top 10 genes frequently observed in cancer patients were *ADCY9*, *RAB3GAP2*, *AURKAPS1*, *EYS*, *SHC2*, *DPP6*, *FREM2*, *ESR1*, *TBC1D22A*, and *ACTN2*. The ten genes frequently observed in non-cancer subjects were *SNTG2*, *LHFPL3*, *DACT2*, *NKAIN2*, *KALRN*, *ABR*, *LMNTD1*, *PLEKHA7*, *DOC2B*, and *ADPRHL1* (Supplementary Table 3). We also studied the prevalence and spectrum of well-known germline cancer susceptibility genes in our subjects [15]. The frequencies of 26 cancer susceptibility genes are shown in Fig. 3b. Deletions in the *FANCA*, *POLD1*, and *STK11* genes were observed in cancer patients only. The frequency of gene deletions was almost the same between cancer and non-cancer subjects. The mutational landscape of DSV genes is shown in Fig. 3c. There were 57 cancer-associated DSV genes with a *p* value < 0.05 in the cancer and non-cancer groups (Supplementary Table 4).

**Fig. 4:**
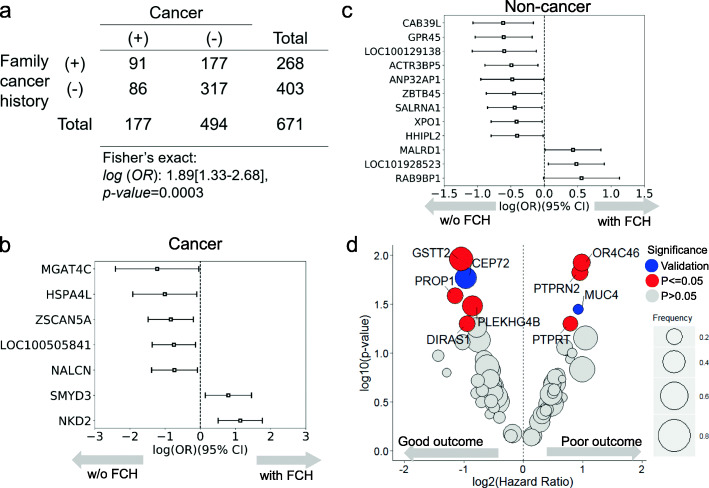
DSV genes and survival analysis in cancer patients with or without family cancer history. **a** Table of the association between cancer and FCH. The subjects who had FCH had a higher risk of developing cancer 1.89 [1.33–2.68] than the subjects without FCH (Fisher’s exact test *p* = 0.0003). The family cancer history is related to first- and second-degree relatives of patients with any cancer. **b** Fisher’s exact test and odds ratio were applied to measure the relationship between each DSV gene and FCH. Forest plot of cancer patients with and without FCH. The DSV genes are *SMYD3* and *NKD2* in cancer patients with FCH. The DSV genes are *MGAT4C*, *HSPA4L*, *ZSCAN5A*, *LOC100505841*, and *NALCN* in cancer patients without FCH. **c** Forest plot of non-cancer subjects with and without FCH. The DSV genes are *MALRD1*, *LOC101928523*, and *RAB9BP1* in non-cancer subjects with FCH. There are nine DSV genes in non-cancer subjects without FCH. **d** Point plot of the log2 hazard ratio DSV genes and log10 (*p* value). The size of the point indicates the frequency of the DSV gene in cancer subjects, and the red marks indicate the eight DSV genes with a *p* value < 0.05. Blue points (*MUC4* and *CEP72* gene deletions) show the validated results

In this study, we found a higher incidence of FCH in cancer patients than in non-cancer subjects (Fig. 4a, *p* value = 0.0003). The study had included 177 cancer patients and 407 non-cancer subjects with family history. Ninety-one of 177 cancer patients presented with family cancer history. One hundred seventy-seven of 407 non-cancer subjects presented with family cancer history. The relationship of cancer and non-cancer subjects with family cancer history was tested by Fisher’s exact test. A higher percentage of cancer patients have a family cancer history (Fig. 4a). The five most cancer types in family-affected members are liver cancer, colorectal cancer, lung cancer, breast cancer, and gastric cancer (Supplementary Table 5).

Moreover, certain DSV genes were associated with cancer or non-cancer subjects with or without FCH (Fig. 4b and c). *MGAT4C*, *HSPA4L*, *ZSCAN5A*, *LOC100505841*, and *NALCN* gene deletions were associated with cancer patients without FCH (*p* < 0.05), while *SMYD3* and *NKD2*DSV genes were associated with cancer patients with FCH (*p* < 0.05). *HHIPL2*, *XPO1*, *SALRNA1*, *ZBTB45*, *ANP32AP1*, *ACTR3BP5*, *LOC100129138*, *GPR45*, and *CAB39L* gene deletions were associated with non-cancer subjects without FCH (*p* < 0.05), while *RAB9BP1*, *LOC101928523*, and *MALRD1* gene deletions were related to non-cancer patients with FCH (*p* < 0.05). Consequently, we inferred that subjects with FCH carrying *SMYD3* or *NKD2* gene deletions may have a higher cancer incidence. As illustrated in Fig. 4d, the volcano plot shows eight significant DSV genes based on the Cox’s proportional hazards model for survival analysis (Supplementary Table 6).

### The clinical impact of immune gene expression-related DSVs in colorectal cancer patients

**Fig. 5:**
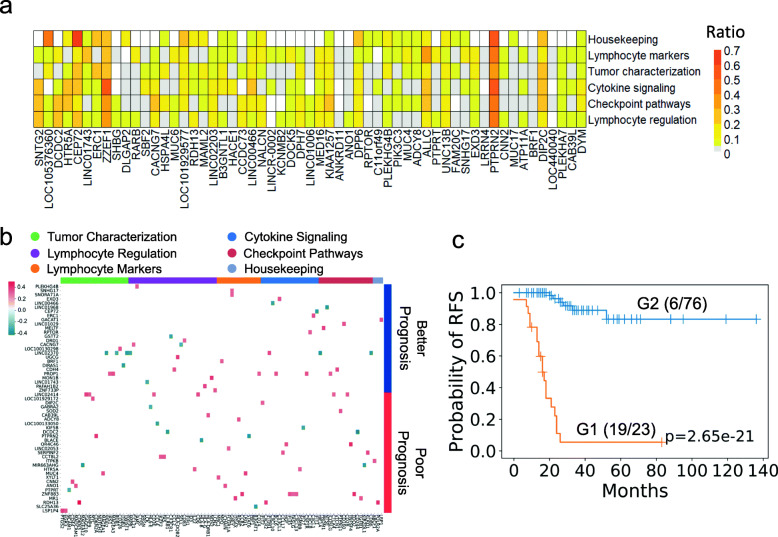
The correlation of DSVs and immune gene expression and prognostic stratification. **a** Heatmap of 57 DSV genes related to six functional immune expression categories. The ratio indicates the frequency of DSV genes related to immune expression. Only two DSV genes, *STNG2* and *LOC105376360*, have *p*-adjusted values < 0.05. **b** Heatmap of 65 immune-related DSV genes and clinical outcomes. There are six functional immune categories: tumor characterization (green), lymphocyte regulation (purple), lymphocyte marker (orange), cytokine signaling (blue), checkpoint pathways (brown), and housekeeping (light blue). The value shown is the point-biserial correlation coefficient in the heatmap. There are poorer prognostic DSV genes correlated with the immune functional tumor characterization category and better prognostic DSV genes related to the immune functional lymphocyte regulation category. **c** RFS by Kaplan–Meier survival plots. The patients were stratified into G1 (orange) and G2 groups (blue) by prognostic deletions that have different clinical outcomes. The G2 group had better clinical outcome than the G1 group

The host immune system differentially participates in the tumor microenvironment. Cancer often develops because of the immune system disturbance caused and functional disorder. The germline DSVs influence aberrant gene expression in tumors [16]. Therefore, we studied the functions associated with 160 immune gene expression-associated DSVs with correlation coefficients of > 0.3, which were selected based on the point-biserial correlation to understand the clinical impact of their deletions (Supplementary Table 1B). There are six categories of immune gene functions: housekeeping, checkpoint pathways, cytokine signaling, lymphocyte markers, lymphocyte regulation, and tumor characterization. A total of 57 DSV genes were correlated with the six functional immune response categories; the *PTPRN2* gene deletion had the highest frequency (Fig. 5a and Supplementary Table 7). *STNG2* and *LOC105376360* gene deletions (Fig. 5a) were related to lymphocyte regulation and housekeeping (*p*-adjusted value less than 0.05), while *CEP72* and *ZZEF1* gene deletions had high occurrences in the housekeeping and cytokine signaling categories, respectively (Fig. 5a).

We selected 65 prognosis-associated DSVs by using survival support vector machine (survival-SVM) [17], which had the highest predicted score for survival-SVM. We used 65 prognosis-associated DSVs among 160 immune-associated DSV genes and constructed a heatmap (Fig. 5b and Supplementary Table 1C). These prognosis-associated DSVs were grouped into poor (33 recurrence-associated DSVs) and better (32 non-recurrence-associated DSVs) prognostic groups using Cox’s proportional hazards model. There were more poor prognostic deletions in the tumor characterization functional category (e.g., *MUC4* and *PTPRN2* gene deletions) and better prognostic deletions in the lymphocyte regulation functional category (Fig. 5b). We then stratified the patients into two groups by prognostic deletions that have different clinical outcomes. Group 1 (G1) was the patient who has more recurrence-associated DSVs than non-recurrence-associated DSVs. According to the Kaplan–Meier curve, these patients in G1 experienced a poor clinical outcome (*p* < 0.05) (Fig. 5c). Patients in group 2 (G2) had better outcomes whose non-recurrence-associated DSVs are more than recurrence-associated DSVs.

### The biological relevance of germline DSVs and tumor microenvironment immune genes

**Fig. 6:**
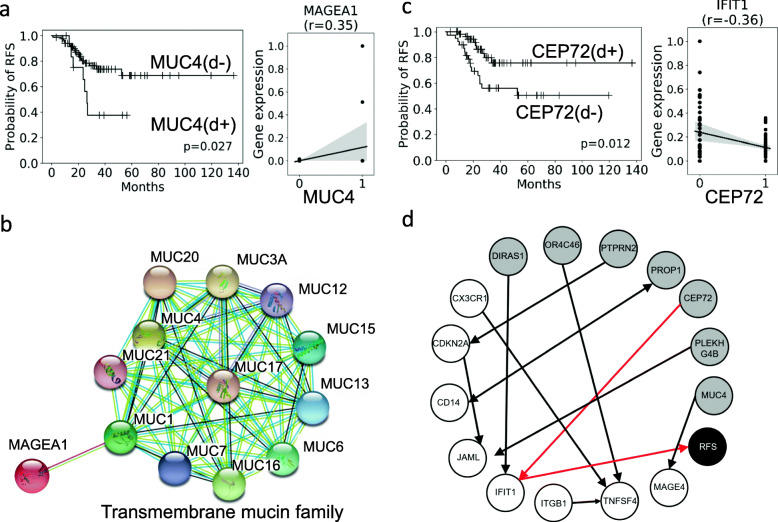
Protein-protein interactions and the causal inference model. **a** Kaplan–Meier survival plot of the *MUC4* gene DSVs. *MUC4* (d−) indicates that cancer patients have no *MUC4* gene deletion. *MUC4* (d+) indicates that cancer patients have the *MUC4* gene deletion. RFS indicates recurrence-free survival. The survival analysis showed that patients with *MUC4* (d−) had a better clinical outcome (*p* = 0.027), and colorectal cancer patients with the *MUC4* gene deletion were associated with increased immune gene (*MAGE1*) expression. The right column shows the *MUC4* gene deletion and *MAGEA1* expression correlation plot (*r* = 0.35). **b** The STRING database was used to show protein-protein interactions of the transmembrane mucin family, including *MUC1*, *MUC4*, and *MAGEA1*. **c** Kaplan–Meier survival plot of *CEP72* DSV. *CEP72* (d−) indicates that cancer patients have no *CEP72* gene deletion. *CEP72* (d+) indicates that cancer patients have a *CEP72* gene deletion. RFS indicates recurrence-free survival. The survival analysis showed that patients with *CEP72* (d+) had a better clinical outcome (*p* = 0.012), and patients with the *CEP72* gene deletion were associated with decreased immune gene (*IFIT1*) expression. The left figure shows the *CEP72* gene deletion and *IFIT1* expression correlation plot (*r* = 0.36). **d** Causal inference model of DSVs, immune gene expression, and RFS. Gray circles represent DSV genes, white circles represent immune expression genes, and the black circle represents RFS. The arrow indicates the causal effect pair. The red arrow pair indicates the RFS causal inference-associated pairs. The causal inference model showed that *CEP72* could affect RFS by *IFIT1*

The tumor microenvironment can affect prognosis and shape therapeutic resistance [18]. Overexpression of the immune *MAGEA1* gene, a member of the *MAGEA* gene family, in tumor and stromal cells is associated with a poor prognosis and an ideal candidate for tumor immunotherapy [19, 20]. *MAGE1* was highly expressed in a previous study on colorectal cancer [20]. In our data, we showed that colorectal cancer patients with germline *MUC4* gene deletion experienced a poor clinical outcome (Fig. 6a). Seven of 13 patients with a germline *MUC4* gene deletion experienced recurrence. Moreover, the *MUC4* gene deletion was positively correlated with *MAGE1* expression, which indicated that SV deletion resulted in increased *MAGE1* expression (Fig. 6a). With the use of the STRING database [21], we also demonstrated protein-protein interactions between the transmembrane mucin family, including *MUC4* and *MAGE1* (Fig. 6b). The functional protein association networks indicated that the *MUC4* gene deletion might influence the expression of *MAGE1*.We hypothesized that germline DSVs could affect immune *MAGEA1* expression and correlate with a poor prognosis.

Here, we also showed that eight prognostic DSVs can affect RFS by expressing tumor microenvironment immune genes. In our cohort, the eight prognostic deletions were correlated with immune gene expression and survival in colorectal cancer stage III patients (Supplementary Fig. 5 and Supplementary Fig. 6). To understand the cause-effect relationship of this result, we applied causal modeling and implemented the PC algorithm by R package CompareCausalNetworks [22]. The PC algorithm uses conditional independence tests for model selection in graphical modeling with directed acyclic graphs [23]. Our results showed that deletion of the oncogene *CEP72* could affect RFS by *IFIT1* immune expression. *IFIT1* is an abundant product of interferon-stimulating genes that correlates with a poor prognosis in cancer [24].

In this study, we demonstrated the possible biological relevance of the *MUC4* gene deletion and *MAGE1* expression and found the causal relationships among *CEP72* gene deletion, *IFIT1*, and RFS (Fig. 6d). *MUC4* is a transmembrane mucin family member, which is expressed in airway epithelial cells and body fluids. *MUC4* plays an important role as a potential candidate for diagnostic and treatment in cancer [25]. *CEP72* is the critical protein for the structural integrity of the centrosome and maintaining microtubule-organizing activity [26]. These results indicate that germline DSVs might affect prognosis by expressing tumor microenvironment immune genes.

## Discussion

Advances in machine learning technologies have led to the use of deep learning prediction models for cancer prevention. Here, we applied WGS of germline DSVs for predicting cancer risk and machine learning methods for assessing immune-related prognosis. Our results highlighted the following: (i) a cancer risk predictive model was established with 671 DSVs and an attention-weighted neural network, (ii) potential markers for inherited cancer risk were identified in cancer patients with or without FCH, (iii) 57 DSVs were correlated with six immune functional categories, (iv) 65 prognostic deletions were identified in order to construct a survival model for clinical outcome stratification, and (v) the possible mechanisms and biological relevance of 2 germline deletions in the expression of two immune genes were presented. Germline WGS and immune gene expression profiling are excellent tools for predicting cancer and stratifying prognosis in colorectal cancer patients.

Traditionally, a small subset of gene alteration features that could predict and classify types of cancer were selected by different machine learning models [27]. However, gene-gene interactions can significantly complicate the search for disease-associated genes. Genes play various essential roles in cancer biology, and each gene carries a different weight importance in the clinical outcome. Deep learning can employ an automatic weight learning feature that can allow complex predictions. In this study, we built a deep learning classification model to identify unique biological features that can differentiate between cancer and non-cancer subjects. Using population-based designs, we identified 671 DSVs associated with the risk of cancer. We found that PCA could distinguish between cancer and non-cancer subjects using these 671 DSVs.

We found that the deletion occur in the *LHFPL3* gene, which is relevant to colorectal cancer. The DSV located at chromosome 7 starts with 104,473,711 end with 104,474,263; the length of DSV was 552 bps. The study has found that *LHFPL3*, the expression of miR-218-5p and miR-138-5p, was downregulated, which correlates to a reduction in cell activity, proliferation, and invasive human ability glioma cells [28]. The deletion in *LHFPL3* leads to gene loss of function, which caused a worsening prognosis in colorectal cancer patients. The DSV in *CBX3* is located at chromosome 7, and the region starts with 26,241,421 ends with 26,245,980. The total length of DSV was 4559 bps (Supplementary Table 1). The result indicated that the deletion in *CBX3* was associated with breast cancer. The Chromobox (CBX) family proteins have epigenetic regulatory function and transcriptionally repress target genes through chromatin modification. The mRNA expression of *CBX3* has been found to affect the outcome of breast cancer in different subtypes. *CBX3* mRNA high expression was correlated to worsening RFS for all breast cancer patients [29].

Many hereditary cancer syndromes have now been defined and attributed to specific germline-inherited mutations. Cancer development is related to accumulating genetic alterations. In this study, we studied the evolution pattern of DSVs in cancer patients with or without FCH. We found that subjects with FCH had a higher incidence of developing cancer and may have initially inherited three DSV genes, namely, *MALRD1*, *LOC101928523*, and *RAB9BP1*. They developed cancer after acquiring two DSV genes: *NKD2* and *SMYD3*. However, patients without FCH may have a different evolution pattern of DSVs. Initially, they inherited nine DSV genes—*CAB39L*, *GPR45*, *LOC1001291138*, *ACTR3BP5*, *ANP32AP1*, *ZBTB45*, *SALRNA1*, *XPO1*, and *HHIPL2*—and developed cancer after acquiring five DSV genes—*MGAT4A*, *HSPA4L*, *ZSCAN5A*, *LOC100505841*, and *NALCN*. We focused on eight signaling pathways associated with the aforementioned DSV genes [30]. The most significant pathway enriched with DSV genes for subjects with FCH was metabolic regulation while for subjects without FCH was transport regulation. These results imply that subjects with or without FCH may develop cancer through different signaling pathways. These DSVs may become useful screening markers.

The result from each classification was the average after five-fold cross validation. The 192 cancer patients and 499 non-cancer samples data were divided into a training set and testing set. We randomly chose 80% samples as the training data and 20% samples as the testing set in each fold. Because the DSV analysis was started with BAM file and lack of samples, there was no other public data can be used as validation data.

Genetic alterations from nature vs nurture: What determines cancer risk and prognosis? We hypothesized that germline DSVs mold the tumor microenvironment and immune gene expression, impacting the clinical outcome. In this study, we wanted to examine the correlation of germline deletions and immune response genes to understand the potential mechanisms by which the tumor microenvironment can affect clinical outcomes [31]. We classified germline structural deletions by the expression of tumor microenvironment-based immune response-associated genes. There were significantly poorer prognostic deletions in the tumor characterization category and better prognostic deletions in the lymphocyte regulation category. Eight prognostic deletions associated with immune gene expression were identified, including *HGF*, *CDKN2A*, and *ITGB1*. They were also reported as poor prognostic factors in a previous study [32].

Beyond the traditional signaling factor statistical survival model, we used the survival-SVM and Cox’s proportional hazards model to select 65 prognostic deletions. We proposed a method to classify risk and non-risk groups by prognostic deletions and identified 57 prognostic DSVs as possible markers for survival stratification and prognosis assessment. From the bioinformatics database and casual inference model, we also demonstrated that immune-associated gene expression may influence the clinical outcome of some germline deletions. The possible mechanism which affects tumor microenvironment survival was shown, but further molecular validation is needed.

## Conclusions

In conclusion, we used genomic data, including WGS and immune gene expression data, and two explainable machine learning models to establish cancer risk predictive models and a prognosis assessment tool that could be useful for cancer prevention and potential therapeutic strategies. Moreover, we need further functional studies to know the biological information of the DSV genes.

## Materials and methods

### Enrollment of cancer patients and non-cancer healthy subjects

This is a hospital-based cohort study of cancer patients. Eligible cancer patients were age ≥ 20 years with histologically confirmed pathological stage II–III adenocarcinoma of colon or rectum, stage II–IV endometrial cancer, I–IV epithelial ovarian cancer, or I–IV breast cancer, an Eastern Cooperative Oncology Group performance status (ECOG PS) of 0–1, and adequate organ function. Patients are willing to provide blood samples for research purposes and written informed consent. Exclusion criteria were receiving chemotherapy within 6 months, other malignancies, and life expectancy less than 1 year. Clinical information, including detailed cancer family history and blood sampling for WGS, health, and lifestyle data of 499 non-cancer normal Taiwanese people ages 30–70, were obtained from Taiwan Biobank.

A total of 192 cancer patients, including eight with breast cancer, 120 with colorectal cancer, 29 with endometrial cancer, and 35 with ovarian cancer, were recruited for the study at the NCKUH between January 2015 and January 2017. Follow-up continued through October 2018. Clinical information (detailed family cancer history (FCH)), tissue, and blood samples for DNA extraction and WGS were collected at the time of enrollment. The NCKUH institutional review board approved this study (A-ER-103-395 and A-ER-104-153), and all participants provided written informed consent. WGS, health, and lifestyle data of 499 non-cancer Taiwanese people were obtained from the Taiwan Biobank as reference (Fig. 1). Of all 99 CRC patients, the distribution of gender was almost the same. The median age of these patients was 58 years. The prevalent primary tumor site was the left colon (80.8%). Family cancer history is related to first- and second-degree relatives of patients with any cancer. Family cancer history and recurrence were not significantly different. There was no significant difference between recurrence and tumor characteristics, such as tumor site, tumor invasion stage (T), or nodal stage (N). In the genetic features of colorectal cancers, there was no significant difference between recurrence and Mismatch Repair (MMR), *KRAS*, and *TP53* status. There were no significant in clinic pathological differentiation and histology features (Table 1).

**Table 1:** 99 CRC patients characteristic

Characteristic	Recurrence (*N* = 25)	Non-recurrence (*N* = 74)	*P* value
Age			0.606
< 65	17 (68%)	55(74.3%)	
≥ 65	8 (32%)	19(25.7%)	
Gender			0.645
Male	11 (44%)	38 (51.4%)	
Female	14 (56%)	36 (48.6%)	
Tumor location			0.576
Left	21 (84%)	55 (74.3%)	
Right	4 (16%)	17 (18.9%))	
Differentiation			0.527
Well	3 (12%)	4 (5.4%)	
Moderate	21 (84%)	66 (89.2%)	
Poor	1 (4%)	4 (5.4%)	
Adenocarcinoma			0.707
Mucinous	2 (8%)	10 (13.5%)	
Nonmucinous	23 (92%)	64 (86.5%)	
Tumor invasion stage			0.733
T1/T2	4 (16%)	9 (12.2%)	
T3/T4	21 (84%)	65 (87.8%)	
Tumor nodal stage			0.292
N0/N1	16 (64%)	57 (77%)	
N2	9 (36%)	17 (23%)	
Family cancer history			> 0.99
Yes	11 (44%)	32 (43.2%)	
No	14 (56%)	42 (56.8%)	
Mismatch repair status			0.325
Proficient	25 (100%)	68 (91.9%)	
Deficient	0 (0%)	6 (8.1%)	
KRAS status			0.3245
Mutated	12 (48%)	25 (34.2%)	
Wild	13 (52%)	48 (65.8%)	
TP53 status			> 0.99
Mutated	22 (88%)	64 (88%)	
Wild	3 (12%)	9 (88%)	

### Study design and workflow

To develop the risk and prognostic stratification model, we collected genomic and clinical information, including FCH, such as survival and FCH, from 192 cancer patients at National Cheng Kung University Hospital (NCKUH) and 499 normal subjects without cancer in the Taiwan Biobank [33] with four aims. First, we aimed to build the cancer risk prediction model with germline DSVs. Second, we studied the spectrum and frequency of DSV genes in cancer patients with or without FCH. Third, we aimed to observe whether genes with DSVs would impact the tumor microenvironment’s immune response gene expression. Fourth, we stratified the cancer patients’ clinical outcomes by immune-related DSVs and investigated the DSVs’ relationship and biological relevance. Figure 1 shows the overall workflow of this study.

### Germline WGS

Genomic DNA from collected blood samples was quantified with a Qubit fluorescence assay (Thermo Fisher Scientific) and sheared with an S2 instrument (Covaris). Library preparation was carried out using the TruSeq DNA PCR-Free HT Kit (Illumina). Individual DNA libraries were measured with 2100 Bioanalyzer (Agilent) qPCR and Qubit (Thermo Fisher Scientific). All flow cells were sequenced on a HiSeq 2500 sequencer (Illumina) using SBS kit V4 chemistry (Illumina). FastQC was used to check read quality, and the resulting reads were aligned to the hg19 reference genome with the BWA-MEM algorithm [34]. The identification of SNPs and indels and genotyping were performed across all samples simultaneously using standard hard filtering parameters or variant quality score recalibration according to GATK Best Practices recommendations [35].WGS was performed with a minimum, median coverage of 30X.

### Immune response gene expression data

Cancer tissues with immune response gene expression profile data were obtained from 99 colorectal cancer patients. RNA was prepared from formalin-fixed paraffin-embedded (FFPE) tissue that was extracted with the RecoverAll Total Nucleic Acid Isolation Kit (Thermo Fisher Scientific). RNA concentration was determined on an Invitrogen™ Qubit™ Fluorometer with the Qubit™ RNA High Sensitivity Assay (Thermo Fisher Scientific). Twenty nanograms of RNA was used for each reverse transcription reaction, and cDNA was prepared with the SuperScript™ IV VILO™ Master Mix Kit. Immune response libraries were prepared using the Ion AmpliSeq™ Kit for Chef DL8 with the Ion Chef™ System and according to instructions in the Oncomine™ Immune Response Research Assay user guide (Pub. No. MAN0015867). The raw gene expression data were preprocessed using Torrent Suite (Thermo Fisher Scientific) and normalized with the min-max feature scaling approach.

### Statistical analysis

The chi-square test and Fisher’s exact test were used to assess the differences between groups. Kaplan–Meier curves were used to evaluate RFS, which was defined as the time between surgery and cancer recurrence. A *p* value < 0.05 was considered statistically significant.

### Machine learning model and analysis

#### Detecting DSVs and data preprocessing

We detected germline DSVs in cancer and non-cancer subjects simultaneously with PopDel from whole-genome DNA sequencing data [10]. DSVs were then filtered by the minor allele frequency (MAF). A MAF greater than or equal to 0.05 and occurring in at least 1% of the sample in each population was subjected to further analysis.

#### Selecting DSVs for the cancer risk and immune expression correlation model

We designed an attention-weighted model [36] to select important DSVs (Fig. 2a). This model is a MLP model based on the attention mechanism. During the learning process, the model automatically adjusts the weight of every DSV. The main aim of this model is to predict subjects with or without cancer. We used the deletion vector for each sample as the input of the attention-weighted model and then adopted binary cross-entropy as a loss function. After training the model, we obtained the weight of each DSV. We then selected cancer risk-associated DSVs with positive weights, which are important when classifying cancer and non-cancer samples. We correlated cancer risk-associated DSVs and immune gene expression data from 99 colorectal cancer patients. The gene expression data were normalized. An immune expression correlation table was established with the point-biserial correlation [37], which was used to correlate continuous variables with dichotomous variables, to determine the relationship between DSVs and immune gene expression.

#### Prognostic candidate genes and survival stratification

There are many survival analyses using the machine learning approach to achieve predicted results, especially survival-SVM [17] which can have better results. We can also know the importance of each DSVs to the model, and it can also be more interpretable. We selected prognosis-associated candidate DSVs by using the survival-SVM [17], which is the approach that can be used to predict the event time duration based on a given set of features. Therefore, we do feature selection base on the survival-SVM, which can select the most predictive prognosis associated with DSVs in the model. The candidate DSVs were clustered into two groups: the recurrence-associated DSV group and the non-recurrence-associated DSV group. We measured the hazard ratio (HR) of each candidate deletion using Cox’s proportional hazards model, which represents the probability of recurrence by giving the survival time of patients. We determined that DSVs with a positive log (hazard ratio) were recurrence-associated deletions, while DSVs with a negative log were non-recurrence-associated deletions. The prognostic DSVs were selected with statistical significance in the hazard model. We used the Kaplan–Meier method for the survival analysis to compare the differences between the two survival curves using the log-rank test [38].

## Supplementary Information

**Additional file 1:.** Supplementary Table 1: DSV information20201208

**Additional file 2:.** Supplementary Table 2: DSV_cluster_pathway_result

**Additional file 3:.** Supplementary Table 3: 20 The frequency spectrum of DSVs

**Additional file 4:.** Supplementary Table 4: 57 The specific genes in subjects

**Additional file 5:.** Supplementary Table 5: FCH cancer types

**Additional file 6:.** Supplementary Table 6: Cox proportional

**Additional file 7:.** Supplementary Table 7: 57 Six functional categories

**Additional file 8:.** Supplementary figures

## Data Availability

The authors confirm that the data supporting the findings of this study are available within the article and its supplementary materials.
