# Supplementary material for: Comprehensive assessments of germline deletion structural variants reveal the association between prognostic MUC4 and CEP72 deletions and immune response gene expression in colorectal cancer patients
Source: Hum Genomics. 2021 Jan 11;15:3. doi: 10.1186/s40246-020-00302-3 (PMC7802320; doi:10.1186/s40246-020-00302-3)
Supplement: Supplementary file 5 — Additional file 5:. Supplementary Table 5: FCH cancer types [file 40246_2020_302_MOESM5_ESM.docx]

| Cancer types | Total(people) |
| --- | --- |
| Liver cancer | 39 |
| Colorectal cancer | 32 |
| Lung cancer | 27 |
| Breast cancer | 27 |
| Gastric cancer | 15 |
| Cervix cancer | 11 |
| Prostate cancer | 10 |
| Head and neck squamous cell carcinomas (HNSCC) | 7 |
| Esophageal cancer | 6 |
| Lymphoma | 6 |
| Pancreas cancer | 5 |
| Nasopharyngeal cancer | 5 |
| Renal cell carcinoma (RCC) | 4 |
| Leukemia | 4 |
| Endometrial cancer | 4 |
| Bladder cancer | 3 |
| Osteogenic sarcoma (OGS) | 3 |
| Thyroid cancer | 3 |
| Skin cancer | 2 |
| Brain cancer | 2 |
| Mutiple myeloma | 2 |
| Aortic tumor | 1 |
| cholangiocarcinoma | 1 |
| Gallbladder cancer (GB) | 1 |
| Small bowel cancer | 1 |

Supplementary table 5. Cancer types in family affected members
