# Supplementary material for: Comprehensive assessments of germline deletion structural variants reveal the association between prognostic MUC4 and CEP72 deletions and immune response gene expression in colorectal cancer patients
Source: Hum Genomics. 2021 Jan 11;15:3. doi: 10.1186/s40246-020-00302-3 (PMC7802320; doi:10.1186/s40246-020-00302-3)
Supplement: Supplementary file 8 — Additional file 8:. Supplementary figures [file 40246_2020_302_MOESM8_ESM.zip › Supplementary figure 1. SV-2020-0820.pdf]

A

	SVM	Logistic Regression	Random Forest	MLP	Attention weighted model
AUC	0.63(+/- 0.02)	0.68(+/- 0.05)	0.58(+/- 0.04)	0.69(+/- 0.03)	0.71(+/- 0.06)
Accuracy	0.78(+/- 0.01)	0.78(+/- 0.01)	0.76(+/- 0.01)	0.79(+/- 0.02)	0.77(+/- 0.04)
Recall	0.29(+/- 0.09)	0.47(+/- 0.07)	0.16(+/- 0.06)	0.46(+/- 0.07)	0.57(+/- 0.12)
Precision	0.81(+/- 0.11)	0.67(+/- 0.07)	0.96(+/- 0.04)	0.69(+/- 0.07)	0.58(+/- 0.07)

B

	SVM	Logistic Regression	Random Forest	MLP	Attention weighted model
AUC	0.81(+/- 0.02)	0.83(+/- 0.04)	0.62(+/- 0.04)	0.78(+/- 0.06)	0.76(+/- 0.06)
Accuracy	0.86(+/- 0.02)	0.87(+/- 0.01)	0.79(+/- 0.01)	0.85(+/- 0.04)	0.82(+/- 0.03)
Recall	0.71(+/- 0.04)	0.75(+/- 0.06)	0.25(+/- 0.10)	0.62(+/- 0.11)	0.62(+/- 0.11)
Precision	0.76(+/- 0.05)	0.78(+/- 0.03)	0.97(+/- 0.05)	0.80(+/- 0.07)	0.70(+/- 0.05)

Supplementary figure 1. The performance of each machine learning strategy.
A. The performance table with 2,919 DSVs. The methods evaluated were the attention-weighted model, multilayer perceptron (MLP), support vector machine (SVM), logistic regression (LR), random forest (RF). Among all methods, the attention-weighted model had the highest area under the curve (AUC) (0.71) and recall rate (0.57).
B. The performance table with 671 cancer-associated DSVs. All of the model performance were increased when using 671 DSVs with positive weights. The AUC of the SVM ranged from 0.63 to 0.86, LR ranged from 0.68 to 0.87, RF ranged from 0.58 to 0.62, MLP ranged from 0.69 to 0.78 and attention-weighted model ranged from 0.71 to 0.76. The DSVs from the attention-weighted model were able to distinguish cancer patients from noncancer subjects.
