# Supplementary material for: Comprehensive assessments of germline deletion structural variants reveal the association between prognostic MUC4 and CEP72 deletions and immune response gene expression in colorectal cancer patients
Source: Hum Genomics. 2021 Jan 11;15:3. doi: 10.1186/s40246-020-00302-3 (PMC7802320; doi:10.1186/s40246-020-00302-3)
Supplement: Supplementary file 8 — Additional file 8:. Supplementary figures [file 40246_2020_302_MOESM8_ESM.zip › Supplementary figure 2. SV-2020-0129.pdf]

A

Supplementary figure 2

Clinical characteristic	Cancer 192(37.8%)	Non-cancer 499 (72.2%)	Total 691(100%)
**Age**			
**Median (range)**	56(23-82)	51(30-70)	53 (23-82)
**Gender**			
**Male**	60 (31.2)	243 (48.7)	303 (43.8)
**Female**	132 (68,8)	256 (51.3)	388 (56.2)
**Place of residence**			
**North**	0 (0)	337 (67.5)	337 (48.8)
**South**	192 (100)	162 (32.5)	354 (51.2)

B

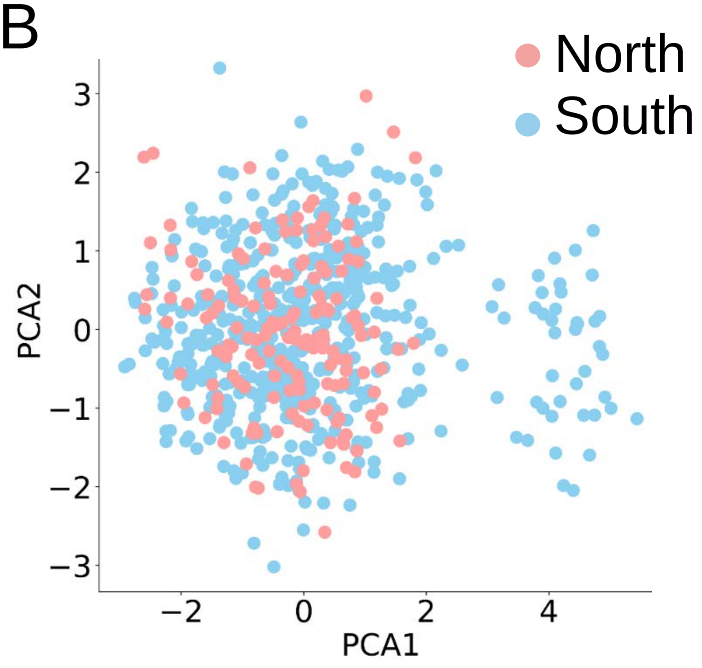

C

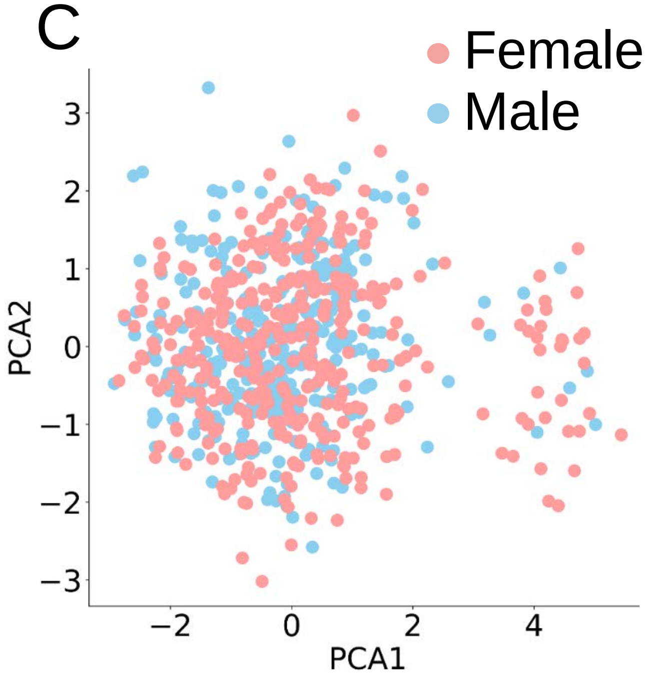

Supplementary figure 2. Demographic characteristics of all subjects

A. Table summary of the demographic features. The study group was composed of 120 colorectal cancer, 29 endometrial cancer, 35 ovarian cancer, and eight breast cancer patients. Females comprised 68.8% of cancer subjects and 51.3% of noncancer subjects. The study group residents all came from southern Taiwan, while 67.5% of the reference group residents came from northern Taiwan.

B. Principal component analysis (PCA) plot of the resident place of all subjects. Red dots indicate residents of northern Taiwan, while blue dots indicate residents of southern Taiwan. There was no population-associated DSV bias factor in the study.

C. PCA plot for the sex of all subjects. Red dots indicate male subjects, and blue dots indicate female subjects. There was no sex-associated DSV bias factor in the study
