# Supplementary material for: Comprehensive assessments of germline deletion structural variants reveal the association between prognostic MUC4 and CEP72 deletions and immune response gene expression in colorectal cancer patients
Source: Hum Genomics. 2021 Jan 11;15:3. doi: 10.1186/s40246-020-00302-3 (PMC7802320; doi:10.1186/s40246-020-00302-3)
Supplement: Supplementary file 8 — Additional file 8:. Supplementary figures [file 40246_2020_302_MOESM8_ESM.zip › Supplementary figure 3. SV-2020-0129.pdf]

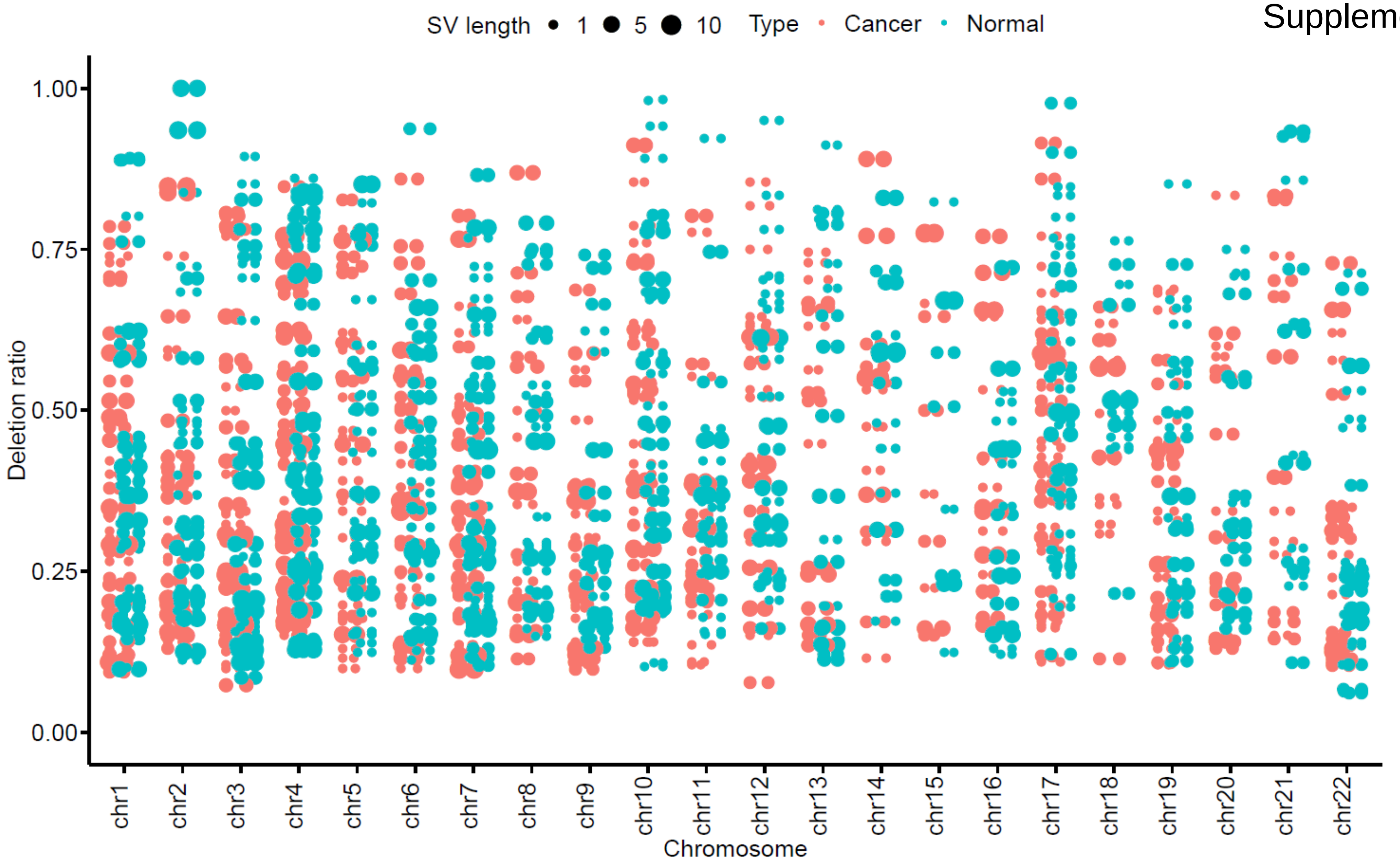

Supplementary figure 3. Percentage distribution of DSVs on each chromosome in cancer and noncancer subjects. Point plot of DSVs in cancer and noncancer subjects. The x-axis indicates the location of the DSVs on the chromosome, and the y-axis indicates the frequency occurrence of DSVs in subjects. Red dots represent cancer subjects, and blue dots represent noncancer subjects. The point size indicates the length of the deletion, ranging from 1 to 10: one indicates a deletion length less than 1000 base pairs (bp), five indicates a deletion length that varies from 4000 to 5000 bp, and ten indicates a deletion length that ranges from 9000 to 10000 bp. The deletion length distribution and chromosome location distribution were not different between cancer and noncancer subjects. Few DSV numbers were observed in chromosomes 13, 14, 15 and 16 in all subjects.
