# Supplementary material for: Comprehensive assessments of germline deletion structural variants reveal the association between prognostic MUC4 and CEP72 deletions and immune response gene expression in colorectal cancer patients
Source: Hum Genomics. 2021 Jan 11;15:3. doi: 10.1186/s40246-020-00302-3 (PMC7802320; doi:10.1186/s40246-020-00302-3)
Supplement: Supplementary file 8 — Additional file 8:. Supplementary figures [file 40246_2020_302_MOESM8_ESM.zip › Supplementary figure 4-2020-0818.pdf]

Supplementary figure 4

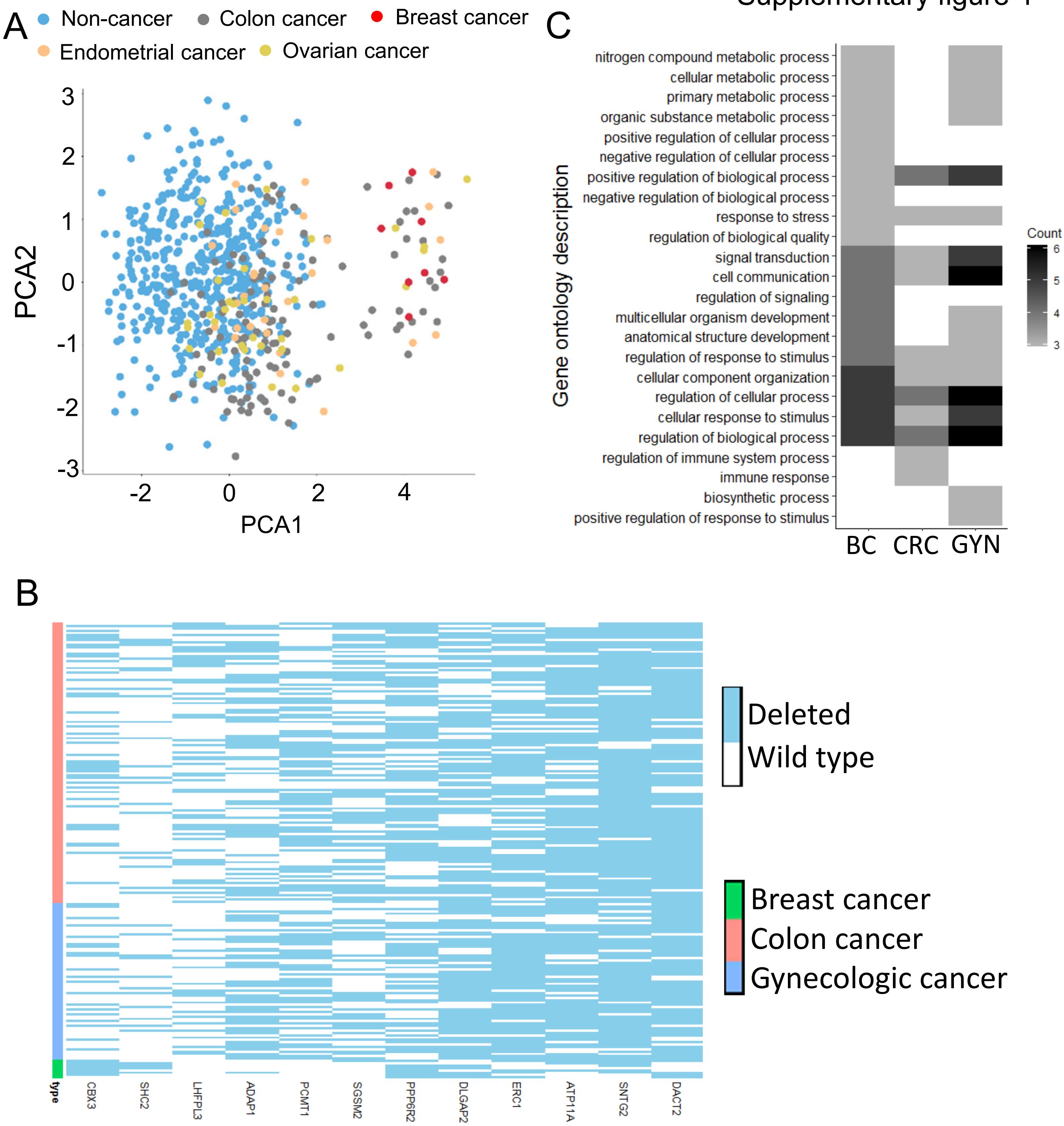

Supplementary figure 4. Cancer type associated DSV genes and signaling pathway analysis

A. Principal components analysis (PCA) plot of all cancer and non-cancer subjects using 671 cancer-associated DSVs. The color label the type of each subject. There is no significant difference in a different cancer type.

B. The heatmap of three types of cancer and 12 DSV genes associated with cancer risk. The cancer-associated DSVs genes are calculated by the odds ratio (>1).

C. The singling pathway of BC, CRC and GYN cancer-associated DSVs genes are annotated with Gene Ontology (GO term) description. (colorectal cancer (CRC), ovarian and endometrial cancer (GYN), breast cancer (BC)) The darkness of blue describes the number of cancer-associated DSVs genes. Most breast cancers are involved with metabolic process signaling pathway. CRC are involved with immune response-related genes signaling pathway.
