# Supplementary material for: Comprehensive assessments of germline deletion structural variants reveal the association between prognostic MUC4 and CEP72 deletions and immune response gene expression in colorectal cancer patients
Source: Hum Genomics. 2021 Jan 11;15:3. doi: 10.1186/s40246-020-00302-3 (PMC7802320; doi:10.1186/s40246-020-00302-3)
Supplement: Supplementary file 8 — Additional file 8:. Supplementary figures [file 40246_2020_302_MOESM8_ESM.zip › Supplementary figure 5. SV-2020-0129.pdf]

Supplementary figure 5

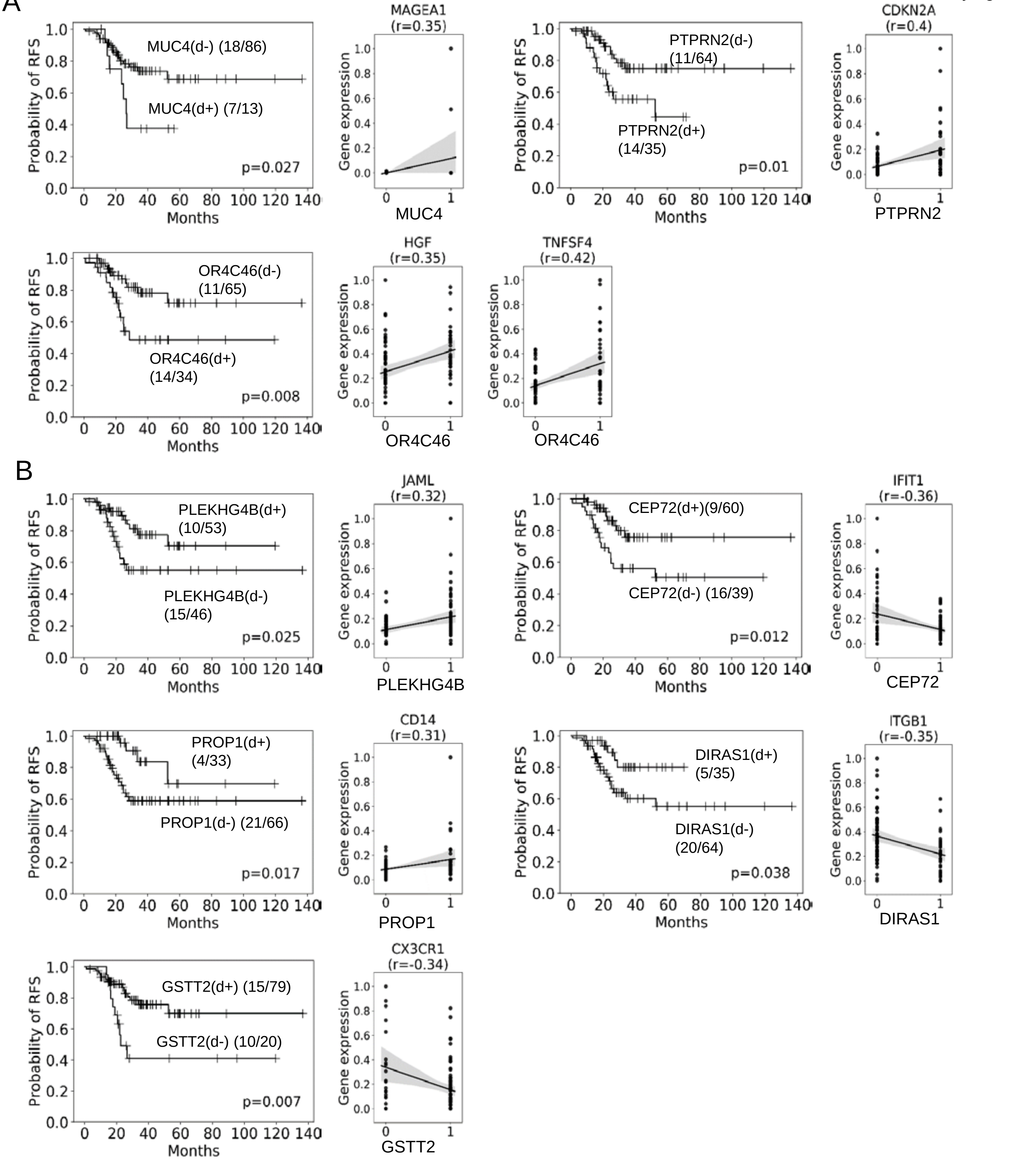

Supplementary figure 5. Recurrence-free survival (RFS) curve and immune gene expression in patients with or without prognostic deletions.

A. DSV genes (MUC4, PTPRN2, and OR4C46) are associated with poor RFS and immune gene overexpression. (d+) means patients with deletion and (d-) means patients without deletion. The related immune overexpression genes are MAGE1, CDNKN2A, HGF, and TNFSF4.

B. DSV genes (PLEKHG4B, CEP72, PROP1, DIRAS1, and GSTT2) are associated with better survival with different immune gene expression. The immune-related overexpression genes are JAML and CD14. The immune-related low expression genes are IFTF1, ITGB1, and CR3CR1.
