# Supplementary material for: Comprehensive assessments of germline deletion structural variants reveal the association between prognostic MUC4 and CEP72 deletions and immune response gene expression in colorectal cancer patients
Source: Hum Genomics. 2021 Jan 11;15:3. doi: 10.1186/s40246-020-00302-3 (PMC7802320; doi:10.1186/s40246-020-00302-3)
Supplement: Supplementary file 8 — Additional file 8:. Supplementary figures [file 40246_2020_302_MOESM8_ESM.zip › Supplementary figure 6. SV-2020-0129.pdf]

Index	Deletion Region	Gene name	Nearest gene	Deletion size (bp)	Cox P-value	Log(Hazard Ratio) [95% CI]	Cancer MAF	Biobank MAF	Related immune response gene	Immune Category
3112	chr3: 195502351 - 195503004	MUC4	MUC20	653	0.036	0.927 [0.059 - 1.795]	0.070	0.089	MAGEA1 (0.35)	tumor characterization
7580	chr7: 157508851-157509403	PTPRN 2	LOC100 506585	552	0.015	0.959 [0.184 - 1.734]	0.174	0.162	CDKN2A (0.395)	tumor characterization
11064	chr11: 51572161-51575548	-	OR4C46	3387	0.012	0.991 [0.213-1.769]	0.229	0.264	HGF (0.35), TNFSF4 (0.421)	cytokine signaling, checkpoint pathways
4204	chr5:83761-84874	-	PLEKH G4B	1113	0.032	-0.855 [-1.640 - -0.069]	0.315	0.317	JAML (0.318)	lymphocyte regulation
4261	chr5: 650701-651549	CEP72	TPPP	848	0.017	-0.971 [-1.769- -0.173]	0.296	0.336	IFIT1 (-0.358)	cytokine signaling
5033	chr5: 177406741-177407563	-	PROP1	822	0.025	-0.316 [-2.162 - -0.138]	0.195	0.252	CD14 (0.312)	lymphocyte markers
16262	chr19: 2713831-2714451	-	DIRAS1	620	0.049	-0.942 [-1.882 - -0.001]	0.179	0.245	ITGB1 (-0.353)	tumor characterization
17578	chr22: 24276421-24279234	-	GSTT2	2813	0.010	-1.041 [-1.842 - -0.241]	0.317	0.364	CX3CR1 (0.336)	lymphocyte regulation

Supplementary figure 6.Table summary of eight prognostic DSV genes and immune expression information.
Deletions are on chromosome 3,5,7,11,19 and 22. The deletion-intersect genes are UC4, PTPRN2, and CEP72. The deletion lengths range from 552 to 2813 base-pairs (bps). The minor allele frequency (MAF) of cancer patients ranges from 0.07 to 0.317. The MAF of Taiwan biobank's non-cancer subjects ranges from 0.089 to 0.364. Three poor prognostic immune gene expressions were functional tumor characteristics categories.
